# The Lichtenstein technique is being used adequately in inguinal hernia repair: national analysis and review of the surgical technique

**DOI:** 10.1590/0100-6991e-20233655-en

**Published:** 2023-11-18

**Authors:** Bruno Amantini Messias, Pedro Lustre de Almeida, Tania Marcela Sandoval Ichinose, Érica Rossi Mocchetti, Cirênio Almeida Barbosa, Jaques Waisberg, Sergio Roll, Marcelo Fontenelle Ribeiro

**Affiliations:** 1 - Hospital Geral de Carapicuiba, Departamento de Cirurgia Geral - Carapicuiba - SP - Brasil; 2 - Centro Universitário São Camilo, Faculdade de Medicina - São Paulo - SP - Brasil; 3 - Universidade Federal de Ouro Preto, Departamento de Cirurgia, Ginecologia e Obstetrícia e Propedêutica - Ouro Preto - MG - Brasil; 4 - Faculdade de Medicina do ABC, Departamento de Cirurgia - Santo André - SP - Brasil; 5 - Santa Casa de São Paulo, Departamento de Parede Abdominal - São Paulo - SP - Brasil; 6 - Hospital Alemão Oswaldo Cruz, Centro de Hérnia - São Paulo - SP - Brasil; 7 - Sheikh Shakhbout Medical City- Mayo Clinic, Critical Care and Acute Care Surgery - Abu Dhab - Emirados Árabes Unidos; 8 - Pontificia Universidade Católica de São Paulo, Departamento de Cirurgia - Sorocaba - SP - Brasil

**Keywords:** Hernia, Abdominal Wall, Herniorrhaphy, Knowledge, Hérnia Inguinal, Parede Abdominal, Herniorrafia, Conhecimento

## Abstract

**Introduction:**

it is estimated that approximately 20 million people undergo inguinal hernia surgery annually in the world, with the Lichtenstein technique being the most performed surgical procedure. The objective of this study is to analyze the knowledge of the technical principles used in the Lichtenstein technique.

**Method:**

Survey-type intersectional study approved by the research ethics committee of São Camilo University Center (CAAE: 70036523.1.0000.0062). During the research period, 11,622 e-mails were sent to members of the main national surgical societies with research on the technical principles of Lichtenstein surgery. The survey was carried out using an electronic form with 10 multiple-choice questions. The form was answered anonymously on the SurveyMonkey and Google Forms platforms.

**Result:**

744 responses were received to the electronic form. Based on this number of respondents, our survey has a confidence level of 95% with a margin of error of 3.5%. It was observed that there is no standardization of the technique among the majority of responders (53.4%). Many surgeons still perform digital dissection of the spermatic cord (47%). A small number of interviewees (15.2%) performed sutures with absorbable thread in the region of the internal oblique aponeurosis, while more than half (55.2%) continued to perform sutures with non-absorbable thread. Most surgeons use a small overlap or fix the mesh juxtaposed to the pubic symphysis (51%).

**Conclusion:**

Our research identified that a small percentage of respondents adequately know the technical principles of Lichtenstein surgery. The result brings us new insights into the need to review Lichtenstein technique.

## RESUMO

**Introdução:**

estima-se que aproximadamente 20 milhões de pessoas sejam submetidas a cirurgia de hérnia inguinal anualmente no mundo, sendo a técnica de Lichtenstein o procedimento cirúrgico mais realizado. O objetivo desse estudo é analisar o conhecimento dos principios técnicos empregados na técnica de Lichtenstein.

**Método:**

estudo tipo levantamento interseccional aprovado pelo comitê de ética em pesquisa do Centro Universitário São Camilo (CAAE: 70036523.1.0000.0062). Durante o período da pesquisa foram encaminhados 11.622 e-mails aos membros das principais sociedades cirúrgicas nacionais com uma pesquisa sobre os princípios técnicos da cirurgia de Lichtenstein. A pesquisa foi realizada por formulário eletrônico com 10 questões de múltipla escolha. O formulário foi respondido de forma anônima nas plataformas SurveyMonkey e Google Forms.

**Resultado:**

foram recebidos 744 respostas ao formulário eletrônico. Com base nesse número de respondedores, nossa pesquisa apresenta grau de confiança de 95% com margem de erro de 3,5%. Foi observado que não há padronização da técnica entre a maioria dos respondedores (53.4%). Muitos cirurgiões ainda fazem dissecção digital do funículo espermático (47%). Um pequeno número de entrevistados (15,2%) realizam sutura com fio absorvível na região da aponeurose do obliquo interno, enquanto, mais da metade (55,2%) continua fazendo sutura com fio inabsorvível. A maior parte dos cirurgiões utilizam overlap pequeno ou fixam a tela justaposta a sínfise púbica (51%).

**Conclusão:**

nossa pesquisa identificou que uma porcentagem pequena dos entrevistados conhecem adequadamente os princípios técnicos da cirurgia de Lichtenstein. O resultado nos traz novas percepções sob a necessidade de revistar a consagrada técnica de Lichtenstein.

## INTRODUCTION

Hernias have been a subject of interest since the beginnings of surgery and their history is as long as the history of man. The first evidence of a patient with inguinal hernia (IH) was described in 1552 B.C. in ancient Egypt^1^ ^-^ ^3^.

The treatment of IH went through several evolutionary phases until reaching current surgical procedures^2^ ^,^ ^3^. Edoardo Bassini elucidated the anterior anatomy of the inguinal canal in 1884 and presented, in 1887, a safe and effective surgical technique that became known as the Bassini repair^2^. This technique brought great understanding of IH and was the first attempt at its definitive surgical treatment^4^. However, his technique and other surgical procedures of the time, such as those of Halsted and McVay, shared a common disadvantage: tension on the suture line^5^.

Due to the unacceptable recurrence rate and prolonged postoperative pain, the hypothesis of using a foreign material to repair hernias was suggested^3^ ^,^ ^4^. The polypropylene mesh was presented by Usher in 1950 in a series of experimental cases, the first clinical studies being presented by this same author only in the late 1950s^2^ ^-^ ^4^.

Based on the hypothesis of the metabolic and degenerative origin of IH, in addition to the adverse effects of the suture line under tension, the Lichtenstein Institute group coined the term tension-free hernioplasty in 1984^2^ ^-^ ^9^. This tension-free surgical technique, described by Irving Lichtenstein, employed a prosthetic material (polypropylene mesh) that was placed between the layers of the internal and external oblique muscles, thus eliminating the need to use unhealthy tissues for closure of the hernia defect^3^. Thus, based on this technical evolution, the fifth principle of inguinal surgery in the modern era was presented: tension-free repair^3^.

In 1989, after detecting four recurrences with the initial technique, Parviz Amid et al. proposed technical modifications (increased mesh size, interrupted suture in the aponeurosis of the internal oblique muscle, and greater overlap in the pubic symphysis). These modifications improved surgical results and culminated in the current Lichtenstein technique modified by Amid, a world-renowned surgical procedure^6^ ^,^ ^8^ ^,^ ^10^ ^-^ ^12^.

The Lichtenstein technique presents five principles based on the characteristic physiodynamics of the abdominal wall and intra-abdominal pressure. These principles are guided by the change in intra-abdominal pressure and the shrinkage of the mesh in living tissue, which is responsible for its contraction. Most authors describe this mesh shrinkage as approximately 20%^4^ ^,^ ^6^ ^,^ ^13^.

The five principles described by Lichtenstein are: (i) use of a footprint shaped mesh measuring approximately 7.5 x 15cm, with coverage of 2cm medially beyond the pubic symphysis, 3 to 4cm above the Hasselbach ligament and 5 to 6cm laterally to the internal inguinal ring; (ii) crossing the ends of the mesh behind the spermatic cord to avoid lateral recurrence; (iii) suturing the mesh with two separate stitches in the sheath of the rectus abdominis muscle and in the aponeurosis of the internal oblique muscle to avoid damage to the iliohypogastric nerve. The lower edge of the mesh must be sutured to the inguinal ligament with a continuous suture (three to four passes) of a non-absorbable thread to prevent mobilization of the mesh; (iv) keep the mesh slightly relaxed or in a dome shape to contain the protrusion of the transversalis fascia when the patient makes some physical effort; and (v) visualize and protect the three nerves found in the inguinal region: ilioinguinal, iliohypogastric, and genital branch of the genitofemoral^3^ ^,^ ^4^ ^,^ ^6^ ^,^ ^8^ ^,^ ^9^ ^,^ ^13^ ^,^ ^14^.

In addition to these five fundamental principles, other technical steps are extremely important in this technique. Lichtenstein and Amid also describe how to evaluate the femoral canal, treatment of the hernial sac, and prevention of chronic pain^8^ ^,^ ^16^.

The femoral canal should be evaluated via the Bogros space through an opening in the posterior wall for direct hernias or through the opening of the hernial sac in cases of indirect hernias. If a femoral hernia is identified, it can be corrected simultaneously using a triangular extension on the mesh fixed to the Cooper’s ligament^8^ ^,^ ^16^.

The indirect hernial sac must be released from the spermatic cord beyond its neck and inverted or reduced to the abdominal cavity without ligation, as this increases the risk of postoperative pain^8^. Several studies and systematic reviews have identified that reduction of the hernial sac results in a lower rate of chronic postoperative pain, without increasing the rate of complications or recurrences^17^ ^-^ ^19^.

After the evolution of the anatomical knowledge of the inguinal region, neuroanatomical comprehension of this region has gained much notoriety. Adequate understanding of the innervation of the inguinal region is extremely important due to the high risk of chronic pain. This complication can affect up to 69% of patients operated on for this disease^21^ ^,^ ^22^. However, clinically significant or disabling pain can be seen in 12% and 6%, respectively^1^.

The inguinal region, as seen from the anterior view, has three main nerves, the ilioinguinal, the iliohypogastric, and the genital branch of the genitofemoral^21^ ^,^ ^22^. Identification of the three nerves is a fundamental step in all patients undergoing IH surgery to prevent nerve damage during dissection and fixation of the mesh.

The ilioinguinal nerve is the most injured in the anterior approach. Prophylactic neurectomy of the ilioinguinal nerve does not seem to reduce the incidence of chronic pain, and apparently increases loss of local sensation. The international hernia consensus does not routinely recommend prophylactic neurectomy^1^. A systematic review conducted by Cirochi et al. corroborates the recommendation of HerniaGroup^1^. However, it also recommends that a prudent surgeon should discuss with the patient and family the benefits and risks of neurectomy if it is necessary to perform it^23^. Excessive manipulation and elevation of nerves from their natural bed increases the risk of perineural fibrosis and chronic pain. In cases of nerve injuries or nerves that are at risk of entrapment, pragmatic neurectomy should be recommended^24^.

Due to its effectiveness and low recurrence, even in the hands of inexperienced surgeons, the tension-free repair recommended by the Lichtenstein group in 1984 was considered the gold standard surgical technique by the American College of Surgeons (ACS)^3^ ^,^ ^6^. Currently, the main national and international consensus and hernia and abdominal wall societies recommend this technique as the surgery of choice for repairing IH via the anterior approach with mesh^1^ ^-^ ^3^ ^,^ ^5^ ^,^ ^6^ ^,^ ^10^ ^-^ ^12^ ^,^ ^15^.

According to Datasus, in the last five years (January 2017 to December 2022), 722,680 IH surgeries were performed by the Public Unified Health System (SUS) in Brazil, with almost all patients (99.2%) undergoing open surgery. Only 5,814 (0.8%) patients underwent laparoscopy. Therefore, the need to know the technical principles of Lichtenstein surgery is still extremely important in Brazil.

This study aims to analyze the knowledge of the technical and anesthetic principles used in Lichtenstein surgery among members affiliated with the main national surgical societies, revisiting its history, evolution, and technical principles.

## METHOD

This intersectional survey study carried out for descriptive analysis was approved by the Ethics in Research Committee of the Centro Universitário São Camilo, under CAAE number 70036523.1.0000.0062 and opinion number 6.249.678. The study was carried out in accordance with the Declaration of Helsinki guidelines, and we followed the STROBE guidelines.

The main national surgical societies (Brazilian College of Surgeons (CBC), Brazilian College of Digestive Surgery (CBCD), and Brazilian Society of Hernia and Abdominal Wall (SBH) sent their 11,622 members an email with the electronic survey on the technical principles and anesthetics used in Lichtenstein’s surgery to IH repair. The e-mails were sent through the societies’ mailing lists. The researchers did not have access to the participants’ personal data. The questionnaire was prepared based on the technical steps described by Lichtenstein and modified by Amid as presented in the introduction and in the referenced articles^4^ ^-^ ^6^ ^,^ ^8^ ^,^ ^13^ ^,^ ^14^ ^,^ ^16^.

The survey was carried out using an electronic form with 10 multiple-choice questions. The form was answered anonymously on the SurveyMonkey and Google forms platforms. The questions regarded 1- the existence of technical standardization in the service?; 2- the anesthetic technique routinely used in Lichtenstein technique repairs; 3- the use of meshes; 4- the size of the mesh; 5- the innervation of the inguinal region; 6- the release of the spermatic cord; 7- the mobilization of the spermatic cord; 8- fixing of the mesh in the aponeurosis of the internal oblique muscle/rectus abdominis sheath; 9- the mesh overlap in the pubic symphysis region; and 10- the mesh fixation overlap (Table 1). The data received were tabulated on Microsoft Excel for analysis and interpretation of the results. We used the most prevalent variables as reference (Ref.)

**Table 1:** Questions and answers asked to research participants

	N	%	p-value
1- Is there a technical standardization in the service you operate?			
Yes, we follow an established technical standard	347	46.60%	0.01
No, teams members use the technique they are used to with their own modifications	397	53.40%	
2- Which anesthetic technique is routinely used in Lichtenstein repairs?			
Spinal anesthesia	695	93.40%	Ref.
General anesthesia	30	4%	<0.001
Local anesthesia	19	2.60%	0.001
3- Regarding the use of meshes:			
I choose the best mesh for the patient to be operated on, as I have several types of meshes available	216	29%	<0.001
I use the mesh available in the hospital, which is not always the most appropriate for the patient	525	70.60%	Ref.
I do not use mesh in hernia repairs	3	0.40%	<0.001
4- Regarding the size of the mesh:			
I use pre-cut, 6 x 12cm meshes	123	16.50%	<0.001
I use 7 x 15cm meshes	418	56.20%	Ref.
I use meshes smaller than 6 x 12cm	34	4.60%	<0.001
I don't worry about the size of the mesh, as long as it is well positioned in the inguinal region	169	22.70%	<0.001
5- Regarding the innervation of the inguinal region:			
I identify nerves routinely	552	74.20%	Ref.
I don't identify nerves routinely	167	22.40%	<0.001
The location of the nerves does not interfere with my mesh placement	25	3.40%	<0.001
6- Regarding the release of the spermatic cord:			
I use digital release	350	47.00%	Ref.
I use release with atraumatic forceps	266	35.80%	<0.001
I use release with an electric scalpel	128	17.20%	<0.001
7- Mobilization of the spermatic cord is done with:			
Gauze	257	34.50%	<0.001
Surgical towel	63	8.50%	<0.001
Penrose drain	375	50.40%	Ref.
Farabeuf retractor	49	6.60%	<0.001
	N	%	p-value
8- Regarding fixation of the mesh in the aponeurosis of the internal oblique muscle/rectus abdominis sheath:			
I perform 2 stitches with absorbable suture parallel to the innervation	113	15.20%	<0.001
I perform random simple stitches in the region	144	19.40%	<0.001
I perform stitches with non-absorbable suture	411	55.20%	Ref.
I perform continuous suturing in the region	76	10.20%	<0.001
9- Regarding the mesh overlap in the pubic symphysis region:			
I use a 2 cm medial overlap	250	33.60%	0.066
I use a 2 cm inferior overlap	115	15.50%	<0.001
I fix the mesh juxtaposed to the pubic symphysis	284	38.20%	Ref.
I use an overlap of less than 2cm	95	12.80%	<0.001
10- Regarding the mesh fixing overlap:			
I use a 2 cm overlap on the aponeurosis of the internal oblique muscle	98	13.20%	<0.001
I use an overlap of 5 to 6cm lateral to the internal inguinal ring	202	27.20%	<0.001
I use an overlap of 2 to 3cm of the internal inguinal ring	265	35.60%	Ref.
I don't worry about overlap as long as the mesh fits in the medial region	179	24.10%	<0.001

Ref, reference: most prevalent variable.

Based on the type of study, we decided to select a probabilistic sample of participants. We compared relative frequencies (percentages or prevalence) and used the Two Proportions Z test for statistical analysis. We applied the Slovin formula for sample calculation, aiming for a 95% confidence level and a 5% margin of error (expected n=372). We used the software SPSS V26 (2019), Minitab 21.2 (2022), and Excel Office 2010, and adopted p<0.05 as a significance level.

## RESULTS

We received responses from 744 participants. The expected answers to questions 4 to 10 and their respective percentages are highlighted in bold in Table 1. Based on this number of respondents, our survey has a confidence level of 95% with a margin of error of 3.5%, better than expected at the beginning of the study, thus increasing the impact of our research. More than half of the participants (53.4%) reported that there is no standardized technique in the service they work in, and that surgeons use their own modifications. Spinal anesthesia was the anesthetic technique most used (93.4%). As for the use of meshes, 70.6% of respondents use the mesh available in the hospital and opt for 7.5 x 15cm meshes for most patients (56.2%). Most respondents reported routinely identifying the nerves in the inguinal region (74.2%). However, 25.8% were not concerned with the identification of the nerves when fixing the mesh. Digital release was the most used technique to release the spermatic cord (47%), followed by atraumatic release (35.8%) and, finally, release with an electric scalpel (17.2%). Fixation in the rectus abdominis sheath/internal oblique aponeurosis is performed with nonabsorbable suture in 55.2%, while fixation with continuous suture is performed in only 10.2%. More than 50% of respondents fix the mesh juxtaposed to the pubic symphysis or with an overlap of less than 2 cm, and only 27.2% of them use an overlap of 5 to 6 cm from the internal inguinal ring.

## DISCUSSION

The Accreditation and Certification of Hernia Centers and Surgeons (ACCESS), which is a group associated to the European Hernia Society, recognizes the need for training of specialists in abdominal wall surgery. This recommendation is based on the wide acceptance of the increasing complexity of abdominal wall surgeries and the need to know several techniques to individualize the treatment of patients with hernias^26^. The main consensus on hernias also recommends the use of an individualized approach to patients^1^ ^,^ ^12^ ^,^ ^26^ and recognizes the need for the techniques used by surgeons to be mastered^26^.

There is no one technique of choice for all IH^1^. Therefore, mastering several techniques, from the simplest to the most complex, is extremely important to deliver the best possible surgical result to the patient. IH can be approached both anteriorly (Lichtenstein, Shouldice, and Bassini) and posteriorly (laparoscopic and robotic). The choice of which technique to use will be based on available resources, the surgeon’s experience, and factors related to the patient and the hernia^1^ ^,^ ^12^ ^,^ ^16^ ^,^ ^27^ ^,^ ^28^. The anterior approach is massively performed with the Lichtenstein technique^1^ ^,^ ^16^ ^,^ ^27^. 

Approximately 46% of respondents stated that they use a standardized technique for Lichtenstein surgery in their services. However, this standardization apparently does not reflect all the technical principles described and modified by Amid, as a much smaller percentage of research participants use appropriate fixings and overlays (15.2% and 33.6%, respectively). We believe that this lack of technical standardization is due to the learning transmitted between surgeons, as some textbooks used in many services as literature for resident training still present inadequate technical steps that were modified by Amid at the end of the 1990s^29^ ^,^ ^30^. 

The identification of the three nerves in the inguinal region is a fundamental step in the Lichtenstein repair and is intended to prevent nerve injury during dissection and fixation of the mesh. The preservation of the nerves in their natural path, associated with the preservation of the fascia that covers them, prevents iatrogenic injury and contact of the mesh with the nerves^16^. More than 20% of respondents still do not worry about routine nerve identification. In the current context, with almost one million hernia surgeries performed in Brazil in the last five years, the possibility of patients with inguinodynia is extremely relevant, and adequate neuroanatomical knowledge of the region is fundamental for the adequate repair of IH^1^ ^,^ ^12^.

Another important modification of the technique was the change in the way of fixing the upper edge of the mesh in the aponeurosis of the internal oblique muscle/rectus abdominis sheath. It is currently recommended that only two interrupted stitches be placed with absorbable thread in the rectus abdominis sheath and internal oblique aponeurosis. This way of fixing the mesh prevents possible injury or entrapment of the iliohypogastric nerve, especially its intramuscular segment, which passes on average 2cm below the lower border of the internal oblique aponeurosis^8^ ^,^ ^16^ ^,^ ^24.^ More than half of respondents (55.2%) still perform sutures with non-absorbable thread in the region and 76 of them (10.2%) perform continuous sutures. Only 15.2% of research participants use correct fixation, with separate stitches of absorbable thread, worrying about local innervation.

Isolation of the spermatic cord must be performed atraumatically, always visualizing the blue line sign (external spermatic vein), as the genital nerve is in close contact with this vein. Digital dissection (circling and elevating the spermatic cord with the fingers) is very traumatic and should no longer be used. This type of dissection leads to injury to the deep cremasteric fascia, which can lead to perineural injury and exposure of the genital branch to the mesh. Despite current recommendations, almost half (47.0%) of those interviewed continue to perform digital dissection of the spermatic cord and 17.2% perform dissection with an electric scalpel. Mobilization of the spermatic cord with a penrose drain is performed by just over half of those interviewed (50.4%). This manipulation with a penrose drain aims to reduce trauma to the topography of the genital branch of the genitofemoral^29^. All efforts and recommendations must be used to try to avoid or reduce the incidence of inguinodynia, a complication that can severely decrease patients’ quality of life^21^.

There are many risk factors for IH recurrence after anterior repair: surgeon experience, tension in the suture line, surgical technique, material, smoking, hernia size, missed hernias, inadequate supervision of residents, and return to activities^31^ ^,^ ^32^. However, a study published in 1987 identified the main causes of recurrence: inadequate coverage in the region of the pubic symphysis (overlap < 2cm) and too narrow a mesh (3 to 5cm) that did not adequately cover the inguinal region^13^ ^,^ ^33^ ^,^ ^34^. Another well-documented cause of recurrence was the non-crossing of the ends of the mesh in the spermatic cord^12^ ^,^ ^14^. Technical modifications were incorporated into Lichtenstein surgery to reduce the risk of recurrence. In our research, many interviewees (35.6%) still use small overlaps in the region of the internal inguinal ring, with less than 30% using the appropriate overlap according to Lichtenstein’s description. Another aspect that draws attention is that more than 50% of those interviewed place the mesh tightly in place or with an overlap of less than 2cm in the region of the pubic symphysis. Lack of adequate overlap in this region is one of the best-known causes of recurrence.

In the first descriptions of the technique, Lichtenstein described the use of 3 x 8cm meshes to correct direct and indirect hernias^34^. Despite the results presented in 1992 by Parviz Amid et al. showing that this mesh size was not sufficient to prevent recurrence^8^ ^,^ ^16^, recommendations for the use of small meshes can still be seen in textbooks^29^. The Lichtenstein Institute recommends that the mesh used should be sized 7.5 cm x 15cm^8^ ^,^ ^16^ ^,^ ^34^. A little more than half (56.2%) of the interviewees use meshes of the recommended size, while 34 (4.6%) still place small meshes in the inguinal region, and three (0.4%) say they do not use meshes in inguinal hernia repairs at all.

It is well established that the use of prosthetic material in IH surgeries reduces the risk of recurrence^27^ ^,^ ^35^. Despite reducing recurrence, the use of mesh has been linked to chronic pain and foreign body sensation, the latter affecting up to 40% of patients. These complications are due to a foreign body reaction from the material used, which leads to an increase in the inflammatory response and the formation of scar tissue. The foreign body reaction is determined by the type, porosity, and volume of material used^40^. There are several characteristics (pore size, weight, pore effectiveness, strength, and elasticity) and materials found in commercially available meshes, but in a pragmatic way, comparing the mesh by weight and porosity is convenient because they are the most evaluated characteristics^1^ ^,^ ^35^. Porosity is a characteristic that impacts the biological behavior of the mesh and appears to interfere with the formation of fibrotic scar tissue and resistance to infection. Large pore meshes are characterized by larger pores of 1 to 1.5mm^12^. The weight of the fabric depends directly on the weight of the polymer as measured in grams/m^2^ (grammage). They can be divided into ultra-low, low, medium, and high grammage^12^. The so-called lightweight or low-weight meshes generally weigh less than 40g/m^2^ and, therefore, cause less inflammatory reaction and foreign body sensation^12^ ^,^ ^25^ ^,^ ^27^. Several studies recommend the use of low weight meshes in the IH repair using the Lichtenstein technique and these meshes have demonstrated a reduction in the incidence of chronic pain and less foreign body sensation, without increasing the number of recurrences, though^25^ ^,^ ^27^ ^,^ ^35^ ^,^ ^36^. The disadvantages of lightweight meshes are their higher cost and their unavailability in most public services. According to our research, most respondents (70.4%) use the meshes available in the hospital and not necessarily the ones that would be best for patients.

When evaluating published scientific studies, we identified pros and cons for each surgical technique. The incidence of chronic pain, recurrence, and complications are very similar and possibly comparable between various techniques^16^ ^,^ ^22^ ^,^ ^37^ ^-^ ^41^ and, therefore, defining a technique as the gold standard is not a very easy task. Regardless of the technique used, each surgeon’s goal should be a recurrence rate and incidence of chronic pain of less than 1%^22^. However, the academic standard of randomized studies and clinical trials generally does not refer to daily practice^16^ and only after a national registry of patients undergoing IH surgeries will we likely have real data regarding the incidence of chronic pain, recurrence, and complications in Brazil^1^.

The most important message regarding IH surgery is not about which technique is best, but about how we should debate and proceed towards improving training, structured residencies, learning curve, surgeries under the supervision of qualified surgeons, hospitals that offer the adequate number of patients so that surgeons in training can master, refine, and deliver the best surgical results to their patients^16^ ^,^ ^31^.

Open surgery to repair IH remains the most accepted and universally performed technique^42^. It is a surgical procedure that every general surgeon must be qualified and technically capable of performing. In addition to its versatility, it is strongly recommended in cases of inguinal pubic pain syndrome, recurrence after posterior approach, and should be considered in cases of peritoneal dialysis, scars due to pelvic surgery, radiotherapy, among others^1^ ^,^ ^42^ ^,^ ^43^. Furthermore, the tension-free technique does not require expensive or exclusive materials, just a polypropylene mesh and a qualified surgeon. It can be performed in any operating room in the world, at low cost and under local anesthesia. Its results are comparable between supervised residents and experienced surgeons, showing the ease of execution and duplication of the procedure^43^.

Following advances in anatomical knowledge and technical refinement, the Lichtenstein technique fulfills many of the requirements of an ideal repair and has the power to address the public health burden of hernia disease in all settings, with low recurrence rates, chronic pain, and morbidity^16^, as long as its technical principles are followed, and its modifications are incorporated into the surgical procedure. Small advances in surgical results can have a tremendous impact on public health, in addition to benefits for patients^16^.

The limitations of our study include low response rate (only 6.5% of the analyzed population), non-response errors, response reliability, and selection errors, limitations characteristic of survey-type studies and selection of probabilistic samples.

## CONCLUSION

Our research identified that a very small percentage of respondents adequately know all the technical principles used in the Lichtenstein technique. The result of our study brings new insights into the need to revisit this renowned technique to deliver the best surgical results to patients with inguinal hernia in Brazil.
